# Comparative predictive value of preoperative GNRI, PNI, and CONUT for postoperative delirium in geriatric abdominal surgery patients admitted to the ICU

**DOI:** 10.3389/fnut.2025.1669159

**Published:** 2025-10-08

**Authors:** Chulin Chen, Yuanyuan Li, Dandan Zhou, Yang Yang, Li Zhang, Xinying Wang

**Affiliations:** ^1^Research Institute of General Surgery, Jinling Hospital, Affiliated Hospital of Medical School, Nanjing University, Nanjing, China; ^2^School of Nursing, Bengbu Medical University, Bengbu, China

**Keywords:** postoperative delirium, nutritional indices, geriatric abdominal surgery, ICU, prediction model

## Abstract

**Background:**

Postoperative delirium (POD) is a serious complication in geriatric patients admitted to the ICU following abdominal surgery. Malnutrition is a significant modifiable risk factor for POD, yet the comparative predictive value of established nutritional indices—Geriatric Nutritional Risk Index (GNRI), Prognostic Nutritional Index (PNI), and Controlling Nutritional Status (CONUT)—remains unclear in this high-risk population. This study aimed to directly compare these indices to identify the optimal preoperative predictor for POD.

**Methods:**

This single-center retrospective study analyzed 333 patients (≥65 years) admitted post-abdominal surgery to the ICU (from October 2021 to December 2024). POD was diagnosed using CAM-ICU. A clinical prediction nomogram was developed based on significant predictors from the multivariate model. The discriminative ability of preoperative GNRI, PNI, and CONUT scores was compared using receiver operating characteristic (ROC) curves, DeLong’s test for the area under the ROC curve (AUC) differences, along with net reclassification improvement (NRI) and integrated discrimination improvement (IDI) to assess model performance enhancements. Optimal cut-off values were determined by maximizing the Youden index, and corresponding sensitivity, specificity, positive predictive value (PPV), negative predictive value (NPV), and kappa statistics were reported. The study was approved by the Institutional Ethics Committee of Jinling Hospital (Approval No. 2024NZKY-038-02).

**Results:**

Factors identified from multivariable analysis (diabetes mellitus, hypoalbuminemia, reduced total cholesterol) were incorporated into a clinical prediction nomogram, which demonstrated good discrimination (AUC = 0.769, 95%CI: 0.707–0.832, *p*<0.001) and calibration (Hosmer-Lemeshow test *p* = 0.444; Brier score = 0.137). Decision curve analysis confirmed its clinical utility. Among the nutritional indices, the CONUT score demonstrated superior predictive performance (AUC = 0.751, 95% CI: 0.686–0.816, *p*<0.001), significantly outperforming PNI (AUC = 0.673, *p*<0.001) and GNRI (AUC = 0.666, *p*<0.001). At an optimal cutoff of 7.5, CONUT achieved 60.9% sensitivity and 81.1% specificity. However, adding CONUT to the clinical nomogram did not significantly improve the predictive performance compared to the clinical model alone (*p* > 0.05).

**Conclusion:**

We developed a practical nomogram and identified the CONUT score as a valuable preoperative predictor for POD—both demonstrating comparable predictive utility. The CONUT score outperformed PNI and GNRI by integrating key biomarkers (albumin, cholesterol, lymphocytes) into a single metric. Although its components overlap with the clinical model, CONUT offers high specificity and simplicity, making it an efficient tool for rapid preoperative risk stratification.

## 1 Introduction

Postoperative delirium (POD) is an acute neurocognitive disorder characterized by fluctuating attention deficits, disorganized thinking, and altered consciousness (1). It represents one of the most common complications in patients admitted to Intensive Care Unit (ICU). Geriatric patients exhibit heightened susceptibility to POD due to age-related physiological decline, reduced cognitive reserve, and the high prevalence of comorbidities, with incidence rates ranging from 13 to 50% (2, 3). POD is associated with catastrophic clinical consequences, including prolonged mechanical ventilation, functional decline, higher healthcare costs, and elevated long-term mortality (1). Despite its clinical significance, POD remains underdiagnosed and lacks targeted therapeutic interventions, highlighting the critical need for early risk stratification and preventive strategies (1, 4).

Malnutrition—prevalent in 30–60% older ICU patients—is an established modifiable risk factor for POD (5). Its sequelae, including muscle catabolism, immune dysfunction, and neuroinflammation, exacerbate neuronal injury (6). However, the precise mechanisms linking preoperative malnutrition to POD pathogenesis remain incompletely elucidated (7). Traditional nutritional assessments often fail to capture these complexities, prompting the adoption of composite indices: The Geriatric Nutritional Risk Index (GNRI), incorporating albumin, weight, and ideal body weight, quantifies nutrition-related risks specific to older populations (8); The Prognostic Nutritional Index (PNI), calculated from serum albumin and lymphocyte count, reflects immune-nutritional status (9); and the Controlling Nutritional Status (CONUT) score, derived from albumin, cholesterol, and lymphocyte counts, screens for undernutrition (10). Emerging evidence suggests that these indices can offer comparable or superior predictive value for POD compared to isolated biomarkers (e.g., albumin), as they capture a broader spectrum of pathophysiological processes (11–13).

Despite promising findings, significant knowledge gaps persist. Current evidence is fragmented across diverse surgical contexts (e.g., gastric surgery (14), cardiac surgery (15), or hip fracture surgery (16)), which vary considerably in their physiological stress, metabolic demands, and impact on nutritional status. Crucially, there is a lack of studies directly comparing the predictive efficacy of GNRI, PNI, and CONUT within the same cohort, making it difficult to determine the optimal tool for specific patient populations. This gap is particularly relevant for geriatric patients undergoing abdominal surgery, a population characterized by high nutritional risk and susceptibility to POD due to the combined effects of age-related decline, surgical stress, and potential disruption of the gut-brain axis (17–19). Therefore, this study aims to directly compare the preoperative predictive value of GNRI, PNI, and CONUT for POD in a uniform cohort of ICU-admitted geriatric abdominal surgery patients. By identifying the most accurate index in this high-risk population, our findings will provide evidence-based guidance for selecting targeted nutritional screening tools, ultimately informing tailored interventions to improve perioperative care and delirium prevention protocols.

## 2 Materials and methods

### 2.1 Study design and population

This single-center retrospective study analyzed the clinical data of 333 geriatric patients (aged ≥65 years) admitted to the ICU following abdominal surgery between October 2021 and December 2024. Inclusion criteria comprised: (1) age ≥65 years; (2) ICU length of stay ≥48 h; (3) history of abdominal surgery with subsequent postoperative ICU admission. Exclusion criteria were: (1) preoperative or historical diagnosis of central nervous system disorders, including preoperative delirium, Parkinson’s disease, dementia (e.g., Parkinson’s disease dementia, Alzheimer’s disease, Lewy body dementia), stroke within the preceding 6 months, or other relevant central nervous system conditions; (2) death during the ICU stay; (3) chronic corticosteroid therapy; (4) severe preoperative visual or auditory impairment; (5) incomplete clinical data records.

### 2.2 Data collection

All clinical data were extracted from the electronic medical record system. Collected demographic and clinical variables encompassed age, sex, body mass index (BMI), smoking history, alcohol consumption, comorbidities (hypertension, cardiovascular disease, respiratory disease, diabetes mellitus), as well as in-hospital management: type of surgery, surgical approach, duration of surgery, requirement for mechanical ventilation. Preoperative laboratory parameters, assessed 1–2 days prior to surgery, included serum albumin, hemoglobin, platelet, white blood cell, total lymphocyte count (TLC), total cholesterol concentration, D-dimer, C-reactive protein, and creatinine. The selection of these laboratory parameters was based on their established pathophysiological links to delirium and related processes, such as tissue oxygenation, systemic inflammation, and hypercoagulability (20).

GNRI was calculated as [14.89 × serum albumin (g/dL)] + [41.7 × (current body weight / ideal body weight)]. Although the index itself has no theoretical upper limit, it stratifies patients into four risk categories based on the following thresholds: major risk (<82), moderate risk (82 ≤ GNRI<92), low risk (92 ≤ GNRI<98), and no risk (≥98) (8). PNI was derived from [10 × serum albumin (g/dL)] + [0.005 × TLC (/μL)]. Similar to the GNRI, the PNI score lacks a definitive upper bound. Nutritional status was classified using established clinical thresholds as follows: severe malnutrition risk (<35), moderate malnutrition risk (35 ≤ PNI ≤ 38), and normal nutritional status (>38) (9). In contrast, CONUT score has a defined range of 0 to 12. It integrated serum albumin (0/2/4/6 points), total cholesterol (0/1/2/3 points), and TLC (0/1/2/3 points) based on predefined thresholds, with total scores categorizing nutritional status as normal (0–1), mild (2–4), moderate (5–8), or severe (9-12) (10).

### 2.3 Diagnosis of POD

POD was diagnosed using the Confusion Assessment Method for the Intensive Care Unit (CAM-ICU), a well-validated and widely utilized instrument for delirium detection in critically ill patients (21). The CAM-ICU assessment comprises four key features: (1) acute onset or fluctuating course, (2) inattention, (3) disorganized thinking, and (4) altered level of consciousness. Information regarding acute onset/fluctuation was obtained from reliable sources (e.g., clinicians, family members). Inattention manifested as difficulty sustaining focus, while disorganized thinking was evidenced by incoherent or illogical communication. Altered consciousness levels were categorized as alert, vigilant, lethargic, stuporous, or comatose. A definitive CAM-ICU delirium diagnosis required the presence of features 1 and 2, plus either feature 3 or 4. The CAM-ICU assessments were performed by trained nursing staff. Prior to each CAM-ICU assessment, the patient’s level of consciousness was evaluated using the Richmond Agitation-Sedation Scale (RASS) (22, 23). CAM-ICU testing was only conducted on patients with a RASS score of −3 or higher (indicating movement or eye-opening to voice but no eye contact). These assessments were performed twice daily (at approximately 8:00 a.m. during morning rounds and 6:00 p.m. during evening rounds) starting on the first calendar day after surgery (postoperative day 1) and continuing until ICU discharge or transfer. All assessment data (both RASS and CAM-ICU) were prospectively recorded in the electronic medical record system at the time of evaluation, from which they were extracted for this study.

### 2.4 Sample size calculation

Sample size was calculated using MedCalc 23.0 for comparing correlated AUCs (DeLong method) (24). With *α* = 0.05 (two-tailed) and 80% power, we estimated AUCs of 0.76 for CONUT (13), and 0.66 for PNI and 0.63 for GNRI from a prior study (25). Targeting the smallest expected difference (*δ* = 0.10, CONUT vs. PNI) and a 25% POD incidence (26), the minimum required sample size was 288.

### 2.5 Statistical analysis

Data are presented according to variable type. Continuous variables (e.g., age, BMI, duration of surgery, and laboratory parameters) are expressed as mean ± standard deviation (SD) if normally distributed, or as median with interquartile range (IQR; 25th–75th percentile) if non-normally distributed. Categorical variables (e.g., sex, smoking history, alcohol consumption, comorbidities, type of surgery, surgical approach and mechanical ventilation) are presented as frequencies and percentages. The distribution of all continuous variables was assessed using the Kolmogorov–Smirnov test. Based on this assessment, group comparisons (POD vs. Non-POD) were performed using: (a) Student’s *t*-test for normally distributed continuous variables; (b) The Mann–Whitney U test for non-normally distributed continuous variables; (c) The Chi-square test or Fisher’s exact test (for more than 20% of expected cell counts were <5) for categorical variables.

To identify independent predictors of POD, a two-step analytical approach was employed: (a) Univariate analysis: All variables were initially analyzed, and those with a significance level of *p* < 0.05 were selected for further analysis; (b) Multivariable logistic regression: The selected variables (diabetes mellitus, type of surgery, mechanical ventilation, albumin, hemoglobin, white blood cell, total cholesterol concentration, D-dimer, C-reactive protein) were entered into a model to adjust for potential confounders. Collinearity between variables was assessed using the variance inflation factor (VIF) and tolerance.

A prediction nomogram was developed based on the final multivariable model to predict the probability of POD. The performance of the nomogram was evaluated in terms of discrimination, calibration, and clinical utility. Discrimination was assessed using the area under the receiver operating characteristic curve (AUC). Calibration was evaluated using calibration curves with 1,000 bootstrap repetitions, the Hosmer–Lemeshow goodness-of-fit test, and the Brier score. Decision curve analysis (DCA) was applied to quantify the net benefits across different threshold probabilities and evaluate clinical utility.

The predictive performance among the different nutritional indices (GNRI, PNI, CONUT) and the two multivariate models was compared using the DeLong test for the difference in area under the receiver operating characteristic curve (AUC). Additionally, net reclassification improvement (NRI) and integrated discrimination improvement (IDI) were calculated to evaluate the improvement in predictive accuracy between models. A two-tailed *p* value < 0.05 was considered statistically significant for all analyses. All analyses were performed using SPSS (version 25.0) and R (version 4.3.3).

### 2.6 Ethics statement

This research adhered to the ethical principles outlined in the Declaration of Helsinki and received approval from the Institutional Ethics Committee of Jinling Hospital (Approval No. 2024NZKY-038-02; Date: 2024-06-18). Given the retrospective design of this study, obtaining written informed consent from participants was formally waived.

## 3 Results

### 3.1 Baseline characteristics and clinical data

**Figure 1:**
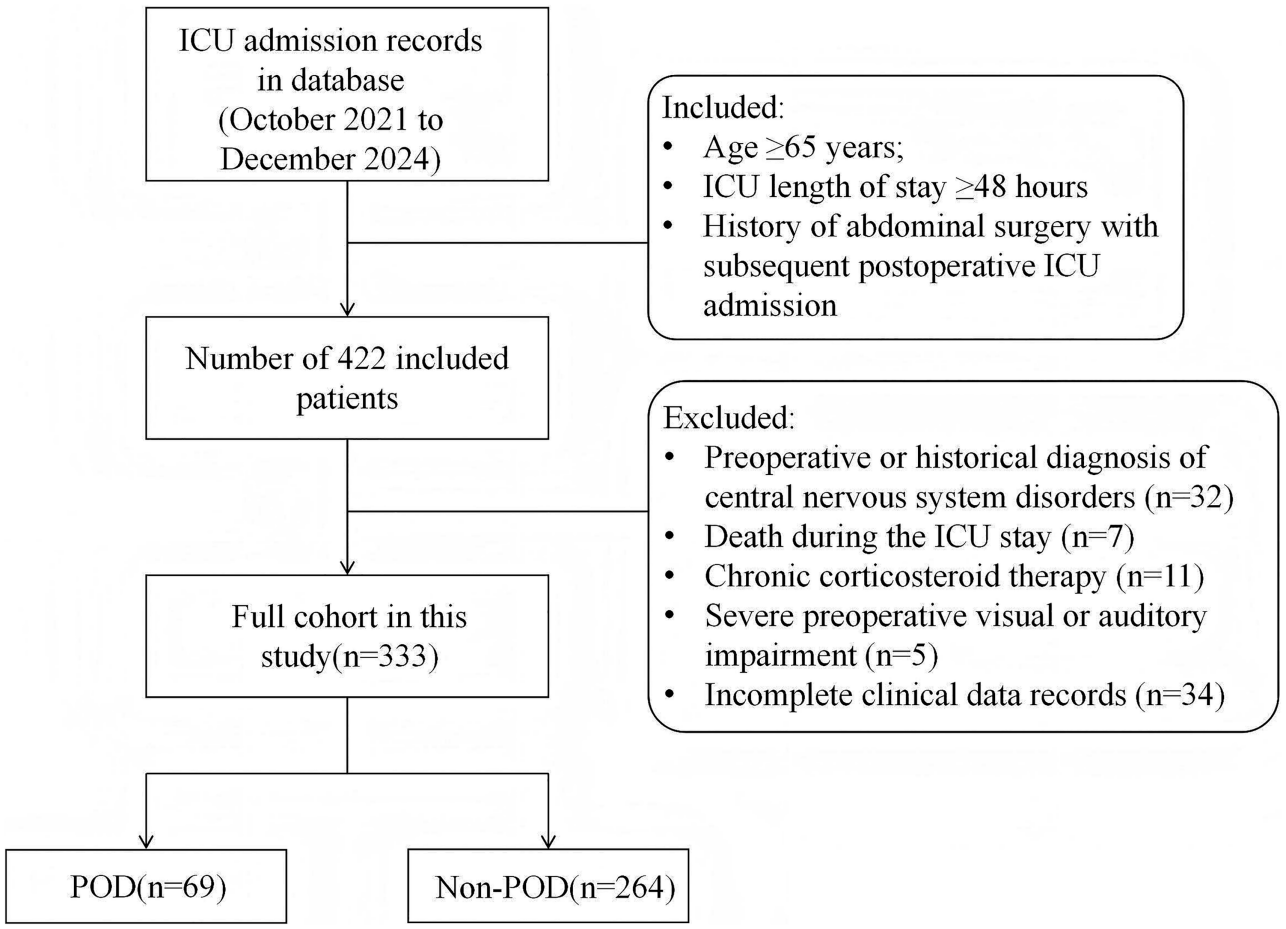
Flow chart of study population.

The detailed selection process could be found in Figure 1. The baseline characteristics and clinical data of patients with POD (*n* = 69) and those without (*n* = 264) are presented in Table 1. No significant differences were observed between the POD and Non-POD groups regarding demographic characteristics. Analysis of comorbidities revealed a significant difference in the prevalence of diabetes mellitus (*p* = 0.002). Significant differences were found in in-hospital management factors and laboratory parameters, including type of surgery, mechanical ventilation, serum albumin, hemoglobin, white blood cell, total cholesterol concentration, D-dimer, and C-reactive protein (*p* < 0.05 for all).

**Table 1:** Comparison of baseline characteristics and clinical data between patients with and without POD.

Variables	POD (*n* = 69)	Non-POD (*n* = 264)	Statistics	*p*-value
Demographic				
Sex [*n*, (%)]			0.472^a^	0.492
Male	49 (71.0)	176 (66.7)		
Female	20 (29.0)	88 (33.3)		
Age [M(P25, P75), year]	75 (69.5, 81)	73 (69, 79)	−1.332^b^	0.183
BMI [M(P25, P75), kg/m^2^]	21.97 (18.7, 25.67)	22.05 (19.56, 25.09)	−0.388^b^	0.698
Smoking history [*n*, (%)]			0.293^a^	0.588
Yes	20 (29.0)	68 (25.8)		
No	49 (71.0)	196 (74.2)		
Alcohol consumption [*n*, (%)]			4.186^a^	0.123
Yes	25 (36.2)	87 (33.0)		
No	44 (63.8)	177 (67.0)		
Comorbidities				
Hypertension [*n*, (%)]			0.876^a^	0.349
Yes	26 (37.7)	116 (43.9)		
No	43 (62.3)	148 (56.1)		
Cardiovascular disease [*n*, (%)]			0.06^a^	0.936
Yes	12 (17.4)	47 (17.8)		
No	57 (82.6)	217 (82.2)		
Respiratory disease [*n*, (%)]			0.234^a^	0.629
Yes	5 (7.2)	24 (9.1)		
No	64 (92.8)	240 (90.9)		
Diabetes mellitus [*n*, (%)]			9.386^a^	0.002^*^
Yes	19 (27.5)	33 (12.5)		
No	50 (72.5)	231 (87.5)		
In-hospital management				
Type of surgery [*n*, (%)]			19.010^a^	<0.001^*^
Emergency	36 (52.2)	66 (25.0)		
Selective	33 (47.8)	198 (75.0)		
Surgical Approach [*n*, (%)]			4.939^a^	0.085
Laparoscopic surgery	18 (26.1)	107 (40.5)		
Robotic surgery	2 (2.9)	5 (1.9)		
Open Surgery	49 (71.0)	152 (57.6)		
Duration of surgery [M(P25, P75), h]	3 (2.15, 4)	2.6 (2, 3.5)	−1.439^b^	0.150
Mechanical ventilation			17.346^a^	<0.001^*^
Yes	30 (43.5)	51 (19.3)		
No	39 (56.5)	213 (80.7)		
Laboratory parameters				
Albumin [(x ± s), g/L]	31.04 ± 5.99	35.53 ± 6.12	5.45^c^	<0.001^*^
Hemoglobin [M(P25, P75), g/L]	109 (84.5, 125.5)	117 (100, 130)	−3.115^b^	0.002^*^
Platelet [M(P25, P75), *10^9/L]	187 (142, 250)	191 (145, 252)	−0.284^b^	0.776
White blood cell [M(P25, P75), *10^9/L]	7.61 (5.695, 10.355)	5.615 (4.5425, 7.9675)	−3.993^b^	<0.001^*^
Total lymphocyte count [M(P25, P75), *10^9/L]	1.01 (0.59, 1.6)	1.18 (0.81, 1.56)	−1.802^b^	0.720
Total cholesterol concentration [M(P25, P75), mm/L]	2.8 (2.07, 3.445)	3.635 (2.9925, 4.405)	−5.968^b^	<0.001^*^
D-dimer [M(P25, P75), mg/L]	2.2 (0.63, 4.62)	1.12 (0.4225, 2.434)	−2.913^b^	0.004^*^
C-reactive protein [M(P25, P75), mg/L]	36.8 (2.9, 122.95)	3.75 (0.7, 28.975)	−4.796^b^	<0.001^*^
Creatinine [M(P25, P75), mg/L]	71.9 (48.2, 115.25)	64.5 (53.95, 79.15)	−1.406^b^	0.160

a Pearson’s chi-square test.

b Mann–Whitney U test.

c Student’s *t*-test.

POD, postoperative delirium.

^*^p<0.05.

**Figure 2:**
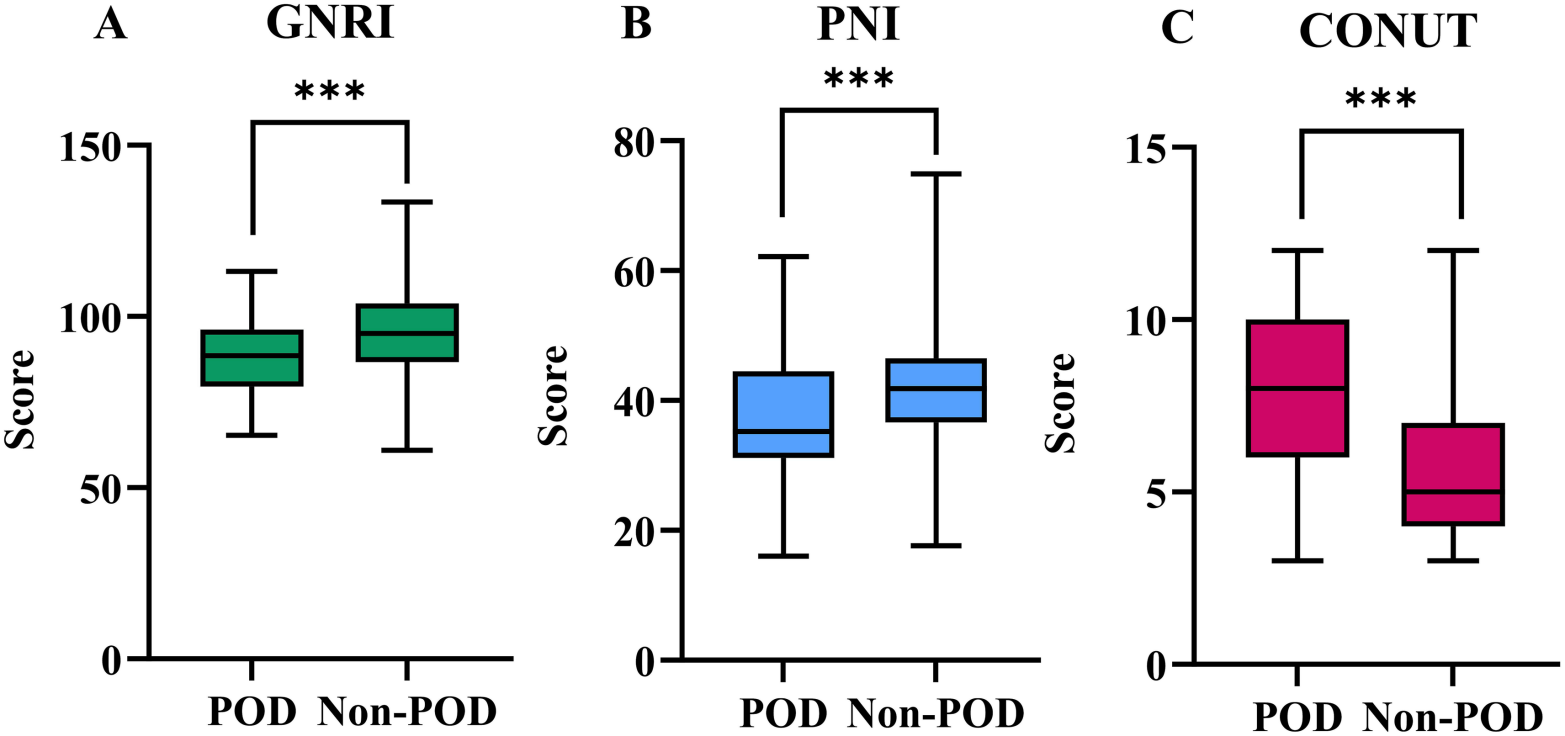
The preoperative values of GNRI **(A)**, PNI **(B)**, and CONUT **(C)** between POD group and Non-POD group (***indicated *p*<0.001).

Figure 2 illustrates the preoperative values of GNRI, PNI, and CONUT between the POD and Non-POD groups. All three preoperative nutritional risk scores showed highly significant differences between the groups (*p* < 0.001 for all). Patients who developed POD had significantly poorer nutritional status as indicated by lower mean GNRI scores (POD: 87.47 ± 11.90 vs. Non-POD: 94.76 ± 12.75), lower median PNI scores (POD: 35.25 [31.18–44.53] vs. Non-POD: 41.85 [36.65–46.5]), and higher median CONUT scores (POD: 8 [6-10] vs. Non-POD: 5 [4-7]) (Supplementary Table 1).

**Figure 3:**
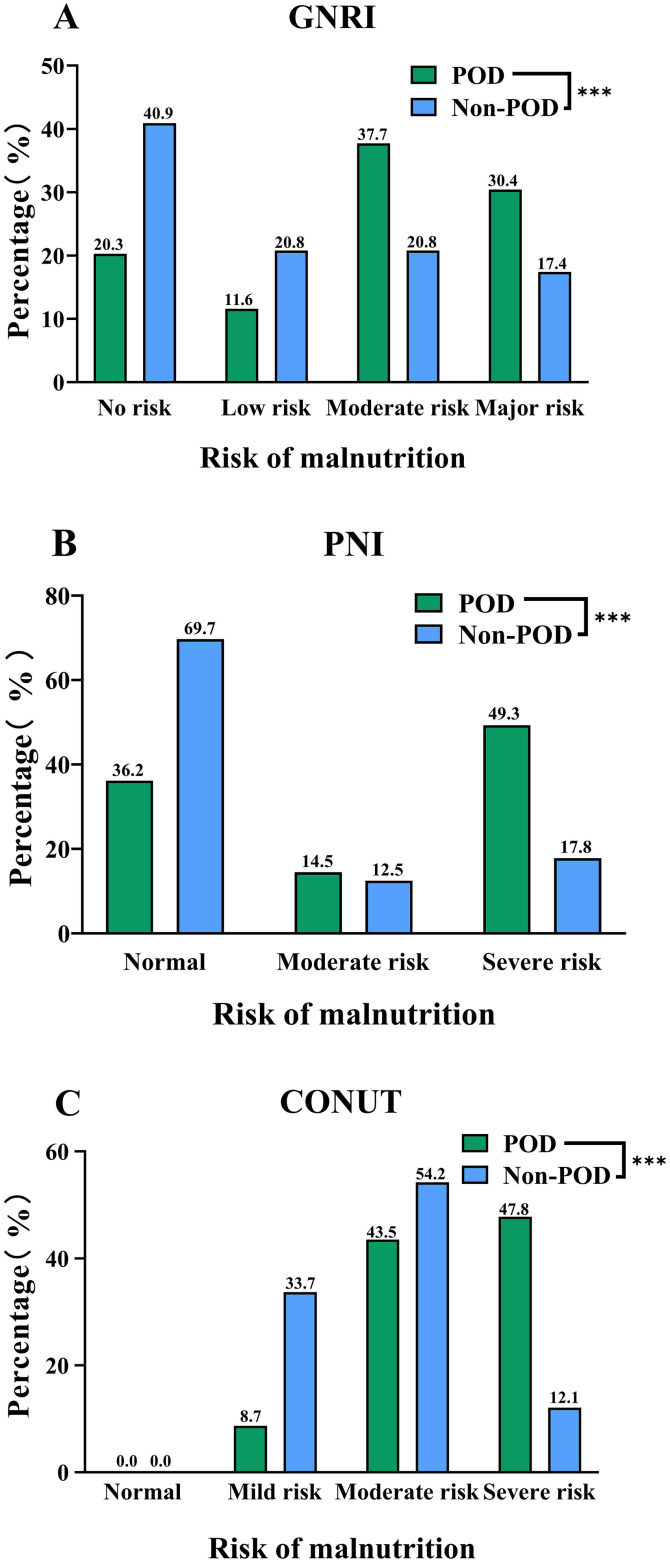
Distribution of GNRI **(A)**, PNI **(B)**, and CONUT **(C)** Categories between POD group and Non-POD group (***indicated *p*<0.001).

Furthermore, Figure 3 illustrates the distribution of patients across nutritional risk categories for each index. According to the GNRI classification (Panel A), a higher proportion of patients in the POD group were categorized with ‘Major’ (30.4% vs. 17.4%) or ‘Moderate’ risk (37.7% vs. 20.8%), whereas the Non-POD group had more patients in the ‘No risk’ category (40.9% vs. 20.3%). Similarly, using the PNI (Panel B), POD was more prevalent among patients with ‘Severe’ (49.3% vs. 17.8%) or ‘Moderate’ risk (14.5% vs. 12.5%), while the Non-POD group showed a higher proportion of ‘Normal’ nutritional status (69.7% vs. 36.2%). Finally, the CONUT index (Panel C) revealed that the majority of POD patients were classified into ‘Severe’ (47.8% vs. 12.1%) or ‘Moderate’ risk (43.5% vs. 54.2%) categories, whereas the Non-POD group had a notably higher percentage in the ‘Mild’ risk category (33.7% vs. 8.7%). All intergroup differences in nutritional risk categories were statistically significant (all *p* < 0.001), as detailed in Supplementary Table 2.

### 3.2 Multivariable logistic regression of factors associated with POD

Variables identified from univariate analysis demonstrated acceptable collinearity diagnostics (tolerance >0.5, VIF < 2; Supplementary Table 3). Subsequent multivariable logistic regression revealed three independent predictors of POD in geriatric abdominal surgery patients: diabetes mellitus (OR = 2.556, 95%CI: 1.232–5.306, *p* = 0.012), hypoalbuminemia (OR: 0.932, 95%CI = 0.879–0.989, *p* = 0.020), and reduced total cholesterol (OR = 0.626, 95%CI: 0.450–0.870, *p* = 0.005) (Full results in Table 2).

**Table 2:** Multivariable logistic regression of factors associated with POD.

Variables	*β*	SE	Wald	*p*-value	OR	95% CI
Diabetes mellitus	0.939	0.373	6.347	0.012^*^	2.556	1.232 ~ 5.306
Type of surgery	0.529	0.383	1.906	0.167	1.697	0.801 ~ 3.596
Mechanical ventilation	0.330	0.379	0.761	0.383	1.392	0.662 ~ 2.924
Albumin	−0.070	0.030	5.444	0.020^*^	0.932	0.879 ~ 0.989
Hemoglobin	−0.006	0.007	0.713	0.398	0.994	0.980 ~ 1.008
White blood cell	0.052	0.032	2.734	0.098	1.054	0.990 ~ 1.121
Total cholesterol concentration	−0.469	0.168	7.756	0.005^*^	0.626	0.450 ~ 0.870
D-dimer	−0.010	0.041	0.064	0.800	0.990	0.913 ~ 1.073
C-reactive protein	<0.001	0.003	0.012	0.911	1.000	0.994 ~ 1.005

*p<0.05; POD, postoperative delirium.

### 3.3 Nomogram of POD risk prediction

**Figure 4:**
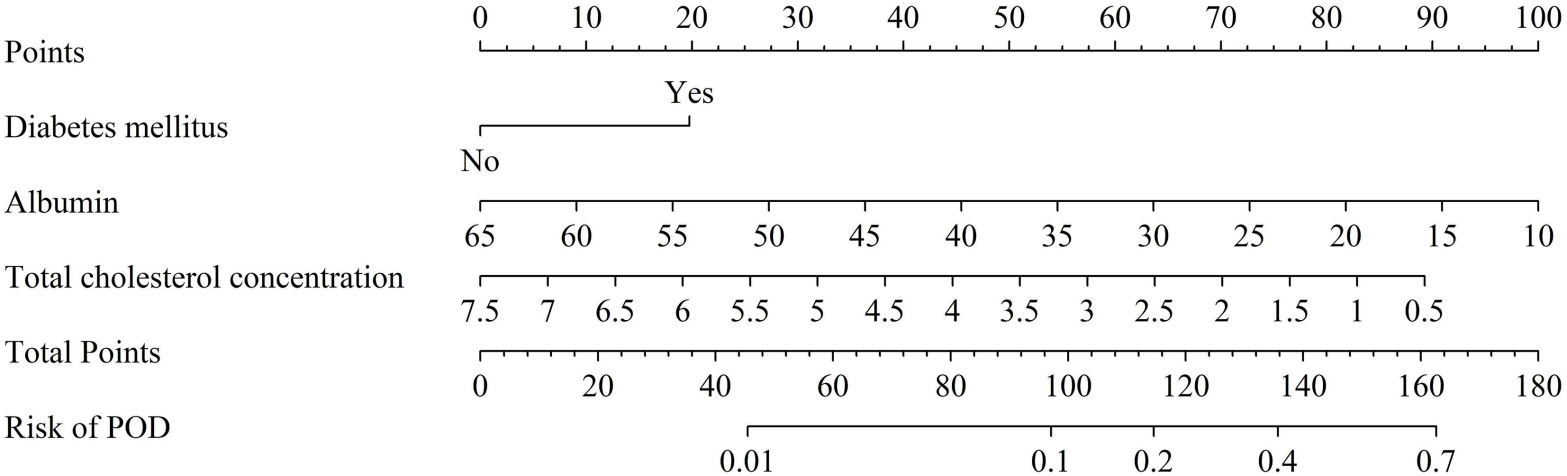
Nomogram of POD risk prediction for geriatric abdominal surgery patients. POD, postoperative delirium.

**Figure 5:**
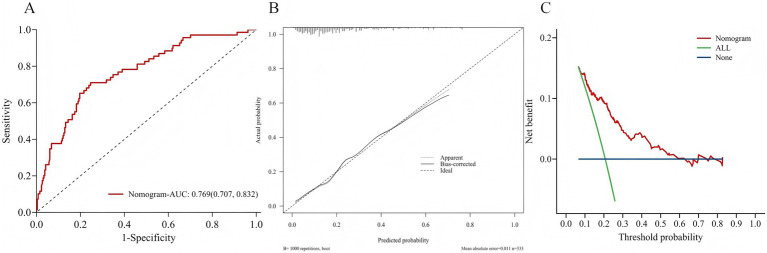
**(A)** Area under the ROC curves for predicting POD. **(B)** The calibration curve for the risk of POD. **(C)** Decision curve analysis of nomogram.

As shown in Figure 4, the three identified independent predictors (diabetes mellitus, albumin, total cholesterol concentration) were incorporated in the nomogram to predicting POD in geriatric abdominal surgery patients. Bootstrap resampling with 1,000 repetitions yielded a C-index of 0.745 (95% CI: 0.742 to 0.749). As shown in Figure 5A, the AUC value was 0.769 with a 95% CI of 0.707–0.832. Figure 5B demonstrates that the nomogram calibration curve for POD probability showed close agreement between the predicted probabilities and actual observations. The Hosmer-Lemeshow test indicated good model fit (χ^2^ = 7.894, *p* = 0.444). The Brier score was 0.137, indicating excellent overall performance. To assess the clinical utility of the nomogram, DCA was conducted as indicated in Figure 5C. According to the decision curve, when the threshold probability for a specific patient is above 0, utilizing the nomogram to predict POD provides greater net benefit compared to either treating all patients or employing no treatment strategy.

### 3.4 Comparative predictive value of different nutritional indices and models for POD

**Figure 6:**
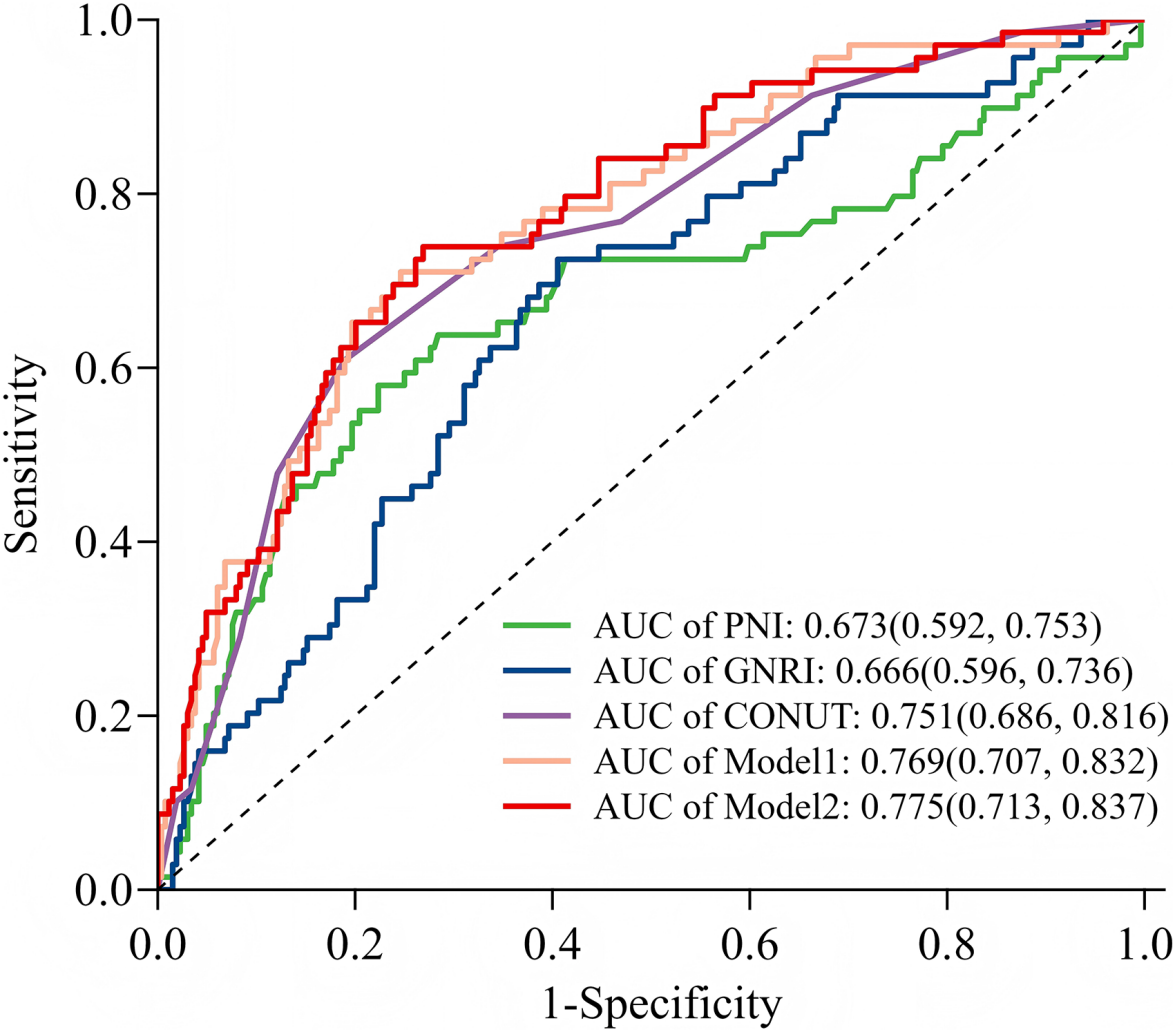
Receiver operating characteristic curve analysis of five models for predicting POD. GNRI, Geriatric Nutritional Risk Index; PNI, prognostic nutritional index; CONUT, Controlling Nutritional Status; Model 1: include diabetes mellitus, albumin, and total cholesterol concentration; Model 2: included CONUT and Model 1.

The predictive performance of the three nutritional indices and the two multivariate models for POD is summarized in Table 3. All tools and models demonstrated significant predictive value, with AUCs exceeding 0.65 (all *p*-values < 0.001). Among the nutritional indices, the CONUT score exhibited the strongest discriminative ability (AUC = 0.751, 95% CI: 0.686–0.816), outperforming both GNRI (AUC = 0.666) and PNI (AUC = 0.673). Both Model 1 and Model 2 (which combined Model 1 with CONUT) also showed excellent predictive performance. At their optimal cut-off values, the CONUT score and the two models achieved a balance between sensitivity and specificity, with Youden indices ranging from 0.420 to 0.558. The ROC curves for all five predictors are visually compared in Figure 6.

**Table 3:** AUC, cut-off value, Youden index, sensitivity, specificity, PPV, NPV and kappa for different nutritional indices and models.

Variables	AUC (95% CI)	*p*-value	Cut-off value	Youden index	Sensitivity (95% CI)	Specificity (95% CI)	PPV (95% CI)	NPV (95% CI)
GNRI	0.666 (0.596, 0.736)	<0.001	93.20	0.320	0.595 (0.535, 0.654)	0.725 (0.619, 0.830)	0.892 (0.846, 0.938)	0.318 (0.246, 0.391)
PNI	0.673 (0.592, 0.753)	<0.001	36.25	0.357	0.777 (0.726, 0.827)	0.580 (0.463, 0.696)	0.876 (0.834, 0.918)	0.404 (0.307, 0.501)
CONUT	0.751 (0.686, 0.816)	<0.001	7.50	0.420	0.609 (0.494, 0.724)	0.811 (0.763, 0.858)	0.457 (0.355, 0.558)	0.888 (0.848, 0.928)
Model 1	0.769 (0.707, 0.832)	<0.001	0.221	0.464	0.710 (0.603, 0.817)	0.754 (0.702, 0.806)	0.430 (0.339, 0.521)	0.909 (0.871, 0.947)
Model2	0.775 (0.713, 0.837)	<0.001	0.205	0.470	0.739 (0.636, 0.843)	0.731 (0.678, 0.785)	0.418 (0.331, 0.506)	0.915 (0.877, 0.952)

Model 1: include diabetes mellitus, albumin, and total cholesterol concentration; Model 2: Model 1 + CONUT. POD, postoperative delirium; PPV, positive predictive values; NPV, negative predictive values; AUC, area under the curve; GNRI, Geriatric Nutritional Risk Index; PNI, Prognostic Nutritional Index; CONUT, Controlling Nutritional Status.

Pairwise comparisons of predictive metrics are detailed in Table 4. The CONUT score significantly outperformed both GNRI and PNI, with statistically greater AUC values (AUC differences: 0.085 and 0.078, respectively; both *p* = 0.001), improved integrated discrimination improvement (IDI), and—in the case of GNRI—better net reclassification improvement (NRI). However, neither Model 1 nor Model 2 showed no statistically significant improvements over the CONUT score alone in terms of AUC or IDI (all *p* > 0.05), although the improvement in NRI was significant for Model 2 versus CONUT (*p* = 0.024). Furthermore, no significant differences were observed between Model 1 and Model 2 in AUC, NRI, or IDI (all *p* > 0.05).

**Table 4:** Performance metrics of different nutritional indices and models to predict POD.

Variables	*Z*	AUC difference (95% CI)	*p*-value	NRI (95% CI)	*p*-value	IDI (95% CI)	*p*-value
GNRI vs. PNI	−0.213	−0.007 (−0.068, 0.055)	0.832	−0.018 (−0.047, 0.011)	0.219	−0.008 (−0.031, 0.014)	0.463
GNRI vs. CONUT	−3.432	−0.085 (−0.133, −0.036)	0.001	−0.089 (−0.167, −0.011)	0.025	−0.090 (−0.123, −0.058)	<0.001
PNI vs. CONUT	−3.305	−0.078 (−0.125, 0.032)	0.001	−0.071 (−0.145, 0.003)	0.060	−0.082 (−0.109, −0.054)	<0.001
Model 1 vs. CONUT	1.009	0.0186 (−0.0175, 0.0547)	0.313	0.163 (0.070, 0.255)	0.001	0.029 (−0.001, 0.060)	0.054
Model 2 vs. CONUT	1.509	0.024 (−0.007, 0.055)	0.131	0.112 (0.015,0.209)	0.024	0.024 (−0.011, 0.060)	0.183
Model 1 vs. Model 2	−0.750	−0.005 (−0.019, 0.009)	0.453	0.050 (−0.007, 0.108)	0.083	0.006 (−0.008, 0.019)	0.409

*p<0.05; Model 1: include diabetes mellitus, albumin, and total cholesterol concentration; Model 2: Model 1 + CONUT. POD, postoperative delirium; AUC, area under the curve; NRI, net reclassification improvement; IDI, integrated discrimination improvement; GNRI, Geriatric Nutritional Risk Index; PNI, Prognostic Nutritional Index; CONUT, Controlling Nutritional Status.

## 4 Discussion

POD has garnered growing recognition as a prevalent perioperative neurocognitive disorder, particularly among elderly surgical patients who are more susceptible to developing this condition (27). Our analysis revealed critical differences between POD and Non-POD groups in geriatric abdominal surgery patients admitted to the ICU. While demographic factors (e.g., age, sex) showed no significant association with POD, diabetes mellitus emerged as a prominent comorbidity. This aligns with existing evidence that hyperglycemia exacerbates neuroinflammation and blood–brain barrier dysfunction, predisposing patients to delirium through microvascular damage and oxidative stress (28). Similar to previous studies (11, 12, 14–16), all three nutritional indices (GNRI, PNI, CONUT) exhibited profound disparities between groups (*p* < 0.001). Patients who developed POD presented with significantly poorer nutritional status, as evidenced by lower GNRI, reduced PNI values, and elevated CONUT scores. These findings underscore malnutrition as a modifiable risk factor for POD, consistent with mechanistic studies linking nutrient deficits to delirium pathogenesis (7, 29). The observed divergence in laboratory parameters (albumin, hemoglobin, cholesterol, C-reactive protein) and clinical management factors (surgery type, mechanical ventilation) between groups further reinforces the complex interplay between metabolic health, systemic inflammation, and surgical stress in POD development (26).

Our multivariable logistic regression identified three independent predictors of POD in this cohort: diabetes mellitus, hypoalbuminemia, and reduced total cholesterol. These findings partially align with prior literature while offering novel insights. Diabetes mellitus consistently elevates POD risk across surgical cohorts, corroborating Xu et al.’s observations in critically ill patients (11). While hypoalbuminemia confirmed Kim et al.’s cardiac ICU findings (13), reduced cholesterol emerged as a distinctive predictor in our cohort—directly contrasting with Kim’s null association in cardiac patients. The interpretation of our findings must be considered in the context of potential unmeasured confounding. Although our analysis controlled for key clinical variables, several important factors known to influence POD risk were not available for adjustment. For example, frailty—a state of heightened vulnerability closely linked to malnutrition—is a powerful independent predictor of delirium (30). Polypharmacy, particularly anticholinergic or sedative medications, can directly precipitate delirium and affect nutritional intake (31). Therefore, it is plausible that the nutritional indices evaluated in our study, particularly CONUT, capture not only nutritional deficits but also the broader physiological dysregulation embodied by frailty and polypharmacy risk. This aligns with established pathways linking malnutrition to POD through impaired immunity, disrupted neurotransmitter balance, and exacerbated stress responses (20).

To simply the prediction process, we developed and validated a multivariable predictive nomogram integrating the three independent predictors identified. This nomogram demonstrated robust performance. Although there are some nomogram models for the prediction of POD in elderly patients (32, 33) and ICU patients (34), no nomogram model for geriatric abdominal surgery patients admitted to the ICU have been developed and evaluated. This tool offers a visualized, quantitative method for clinicians to estimate individual POD risk preoperatively, thereby facilitating personalized monitoring and potential early intervention strategies.

Early POD prediction enables timely intervention in high-risk patients. While the prognostic value of nutritional indices is well-established in the literature, the existing evidence remains fragmented across studies focusing on single indices within specific populations. For instance, PNI has been inversely associated with POD incidence in critically ill patients (11), elderly spinal surgery patients (35), and those undergoing hip fracture surgery (36), total hip arthroplasty (32), or noncardiac surgery (37). Similarly, studies focusing solely on GNRI have shown that higher values decrease POD risk in elderly ICU patients (12), cardiac surgery patients (15), those with degenerative lumbar disease (38), and patients undergoing gastric (14) or non-cardiac surgery (39). Hu et al. further confirmed that other albumin-derived markers (NAR, PNI, SIS) predict POD in elderly total hip arthroplasty patients (32). This siloed approach makes it difficult to determine the relative predictive power and optimal choice of nutritional index for clinical use.

Our study addresses this critical gap by providing the first direct comparative analysis of three established nutritional indices within a unified cohort of ICU-admitted geriatric abdominal surgery patients. Our findings demonstrate that while all indices are significant predictors, the CONUT score exhibited superior discriminative capacity (AUC = 0.751), outperforming both PNI (AUC = 0.673) and GNRI (AUC = 0.666). This partly aligns with Kim et al.’s findings in cardiac ICU cohorts but extends applicability to abdominal surgery—a population with distinct nutritional challenges due to gut dysfunction and surgical stress (13). Their analysis revealed fair delirium-predictive performance for these indices (AUC: GNRI = 0.729, PNI = 0.728, CONUT = 0.762, *p*>0.05). We hypothesize that CONUT’s superiority stems from its multidimensional design, which integrates albumin, cholesterol, and lymphocytes. This comprehensive approach may be particularly advantageous in abdominal surgery patients, where we propose the gut-brain axis plays a critical role (17–19). Abdominal surgery directly causes gut dysfunction, potentially amplifying systemic inflammation and disrupting neurotransmitter synthesis. The CONUT score, by incorporating cholesterol (a precursor for neuroactive steroids) and lymphocytes (a marker of immune competence), may better capture this diet-modulated, gut-brain pathophysiology than indices relying on fewer parameters.

At the optimal cutoff of 7.5, the CONUT score demonstrated high specificity (81.1%) valuable for minimizing false positives, though with moderate sensitivity (60.9%). The multivariable Model 1 (incorporating diabetes mellitus, albumin, and total cholesterol) showed comparable discriminative ability to CONUT in terms of AUC and IDI (both *p* > 0.05), but provided a statistically significant improvement in net reclassification (*p* = 0.001), indicating similar overall predictive performance between the comprehensive clinical model and the simpler CONUT tool. The high specificity of CONUT supports its utility in “ruling in” high-risk cases, suggesting a rational two-stage screening strategy analogous to established nutritional assessments (40). As an objective, rapid tool based on routine parameters (albumin, cholesterol, lymphocytes), CONUT serves as an efficient first-line screener. Patients identified as high-risk can then undergo further evaluation using the full nomogram. This approach leverages the strengths of each method: CONUT’s simplicity and specificity for initial triage, and the nomogram’s integrative capacity for definitive stratification.

Notably, integrating the CONUT score into the clinical model (Model 2) did not yield statistically significant improvements in AUC, NRI or IDI compared to Model 1 (*p* > 0.05), indicating that the predictive information conveyed by CONUT is largely encompassed by its components-albumin and total cholesterol-within a multivariate framework. This statistical overlap underscores the clinical importance of both hypoalbuminemia (reflecting inflammation and protein catabolism) and hypocholesterolemia (indicating malnutrition and metabolic dysfunction) in POD pathogenesis (41, 42). The CONUT score effectively synthesizes these key elements into a single metric, maintaining its value as a practical, standalone preoperative screening tool despite limited incremental value in combined modeling. By consolidating essential biomarkers into an easily calculable score, CONUT provides clinicians with a rapid, holistic nutritional risk assessment without complex modeling requirements, particularly beneficial in resource-limited settings (43) for translating nutritional risk assessment from research to bedside practice.

## 5 Limitation

This study has several limitations. First, the single-center retrospective design may introduce selection bias; prospective validation in diverse populations is warranted. Second, the overall sample size is limited, and the significant imbalance between the POD (*n* = 69) and Non-POD (*n* = 264) groups may affect the precision of our estimates and the performance of the multivariable model. Third, unmeasured confounders (e.g., frailty, intraoperative anesthesia type, sedative use, opioid dosage) could affect nutritional-POD relationships. Finally, we did not account for perioperative nutritional support, which might confound associations between nutritional indices and POD. Therefore, future multi-center prospective studies addressing these limitations are essential.

## 6 Conclusion

In conclusion, we developed a practical nomogram (using diabetes, albumin, and total cholesterol) and identified the CONUT score as a valuable preoperative predictor for POD—both demonstrating comparable predictive utility. The CONUT score outperformed PNI and GNRI by integrating key biomarkers (albumin, cholesterol, lymphocytes) into a single metric. Although its components overlap with the clinical model, CONUT offers high specificity and simplicity, making it an efficient tool for rapid preoperative risk stratification.

## Data Availability

The raw data supporting the conclusions of this article will be made available by the authors, without undue reservation.
