# Supplementary material for: Comparative predictive value of preoperative GNRI, PNI, and CONUT for postoperative delirium in geriatric abdominal surgery patients admitted to the ICU
Source: Front Nutr. 2025 Oct 8;12:1669159. doi: 10.3389/fnut.2025.1669159 (PMC12540140; doi:10.3389/fnut.2025.1669159)
Supplement: Supplementary file 1 [file Table_1.docx]

Supplementary Material

**Supplementary Table 1 The preoperative values of GNRI, PNI, and CONUT between POD group and Non-POD group**

| Preoperative scores | POD（n=69） | Non-POD（n=264） | Statistics | P-value |
| --- | --- | --- | --- | --- |
| GNRI [(x̄±s)] | 87.47±11.90 | 94.76±12.75 | 4.288^a^ | ＜0.001 |
| PNI [M(P25, P75)] | 35.25(31.18, 44.53) | 41.85(36.65, 46.5) | -4.416^b^ | ＜0.001 |
| CONUT [M(P25, P75)] | 8(6, 10) | 5(4, 7) | -6.481^b^ | ＜0.001 |

a: Student’s t-test; b: Mann-Whitney U test

**Supplementary Table 2 Distribution of GNRI, PNI, and CONUT categories between POD group and Non-POD group**

| Preoperative scores | POD（n=69） | Non-POD（n=264） | Statistics | P-value |
| --- | --- | --- | --- | --- |
| GNRI |  |  | 19.802^a^ | ＜0.001 |
| No risk | 14(20.3) | 108(40.9) |  |  |
| Low risk | 8(11.6) | 55(20.8) |  |  |
| Moderate risk | 26(37.7) | 55(20.8) |  |  |
| Major risk | 21(30.4) | 46(17.4) |  |  |
| PNI |  |  | 32.205^a^ | ＜0.001 |
| Normal | 25(36.2) | 184(69.7) |  |  |
| Moderate risk | 10(14.5) | 33(12.5) |  |  |
| Severe risk | 34(49.3) | 47(17.8) |  |  |
| COUNT |  |  | 48.930^a^ | ＜0.001 |
| Normal | 0 | 0 |  |  |
| Mild risk | 6(8.7) | 89(33.7) |  |  |
| Moderate risk | 30(43.5) | 143(54.2) |  |  |
| Severe risk | 33(47.8) | 32(12.1) |  |  |

a: Pearson’s chi-square test

**Supplementary Table 3 The tolerance and VIF for diabetes mellitus, type of surgery, mechanical ventilation, albumin, hemoglobin, white blood cell, total cholesterol concentration, D-dimer and C-reactive protein**

| Items | Tolerance | VIF |
| --- | --- | --- |
| Diabetes mellitus | 0.969 | 1.032 |
| Type of surgery | 0.588 | 1.700 |
| Mechanical ventilation | 0.732 | 1.366 |
| Albumin | 0.687 | 1.455 |
| Hemoglobin | 0.785 | 1.274 |
| White blood cell | 0.821 | 1.218 |
| Total cholesterol concentration | 0.748 | 1.337 |
| D-dimer | 0.702 | 1.425 |
| C-reactive protein | 0.565 | 1.771 |

VIF, variance inflation factor
